# Fractal dimension approach in postural control of subjects with Prader-Willi Syndrome

**DOI:** 10.1186/1743-0003-8-45

**Published:** 2011-08-20

**Authors:** Veronica Cimolin, Manuela Galli, Chiara Rigoldi, Graziano Grugni, Luca Vismara, Luca Mainardi, Paolo Capodaglio

**Affiliations:** 1 Bioengineering Department, Politecnico di Milano, p.zza Leonardo Da Vinci 32, 20133, Milano, Italy; 2 Orthopaedic Rehabilitation Unit and Clinical Lab for Gait Analysis and Posture, Ospedale San Giuseppe, Istituto Auxologico Italiano, IRCCS, Via Cadorna 90, I-28824, Piancavallo (VB), Italy; 3 IRCCS "San Raffaele Pisana", Tosinvest Sanità, Roma, Italy; 4 Unit of Auxology, Ospedale San Giuseppe, Istituto Auxologico Italiano, IRCCS, Via Cadorna 90, I-28824, Piancavallo (VB), Italy

## Abstract

**Background:**

Static posturography is user-friendly technique suitable for the study of the centre of pressure (CoP) trajectory. However, the utility of static posturography in clinical practice is somehow limited and there is a need for reliable approaches to extract physiologically meaningful information from stabilograms. The aim of this study was to quantify the postural strategy of Prader-Willi patients with the fractal dimension technique in addition to the CoP trajectory analysis in time and frequency domain.

**Methods:**

11 adult patients affected by Prader-Willi Syndrome (PWS) and 20 age-matched individuals (Control group: CG) were included in this study. Postural acquisitions were conducted by means of a force platform and the participants were required to stand barefoot on the platform with eyes open and heels at standardized distance and position for 30 seconds. Platform data were analysed in time and frequency domain. Fractal Dimension (FD) was also computed.

**Results:**

The analysis of CoP vs. time showed that in PWS participants all the parameters were statistically different from CG, with greater displacements along both the antero-posterior and medio-lateral direction and longer CoP tracks. As for frequency analysis, our data showed no significant differences between PWS and CG. FD evidenced that PWS individuals were characterized by greater value in comparison with CG.

**Conclusions:**

Our data showed that while the analysis in the frequency domain did not seem to explain the postural deficit in PWS, the FD method appears to provide a more informative description of it and to complement and integrate the time domain analysis.

## Background

Balance is a key function for performing daily life tasks. In the evaluation of patients complaining of balance disorders, postural instrumental analysis plays nowadays an increasingly important role. Instrumental analysis can indeed add to clinical examination quantitative information on balance ability. Whereas clinical examination provides insight into the physiopathology and aetiology of the disorder and functional scales rate its severity and the related risk of fall, instrumental evaluation can provide objective baseline and outcome measures for evidence-based rehabilitation programs. In particular, static posturography has been extensively used in populations of various age to study the biomechanical effects on gross motor skills in subjects affected by various motor disorders (Cerebral Palsy, Muscular Dystrophy, spinal cord injuries), fine cognitive or learning disabilities (autism, Developmental Coordination Disorder, Attention Deficit Hyperactivity Disorder and dyslexia) [1], genetic disorders (Down syndrome, Prader-Willi syndrome) [2,3] and obesity [4]. Platform stabilometry is the measurement of forces exerted against a force platform during quiet stance, commonly used to quantify the body sways of an individual in a standing position. It is widely used in clinical settings to obtain functional markers on fine competencies and their development and a large number of posturographic measures are sensitive to testing condition (i.e. eyes open vs. eyes closed, feet position, and presence of external stimuli).

Static posturography is user-friendly and suited for the analysis of the center of pressure (CoP) trajectory (length, surface, maximal amplitude of the displacement, speed, and frequency analysis) in everyday practice. However, the information obtained cannot be univocally interpreted from a physiological point of view. The CoP is in fact a measure of whole body dynamics and thereby represents the sum of various neuro-musculoskeletal components acting at different joints level. In addition the CoP time series is two dimensional or planar because it represents the reaction forces on the supporting surface. Although the two components of the signal, anterior-posterior and medio-lateral, are often analysed separately, they represent the output of a unique integrated system. As a consequence, the utility of static posturography in clinical practice is somehow limited and there is a need for reliable approaches in order to extract physiologically meaningful information from stabilograms [5-7].

Recently, some advanced mathematical methods have been proposed to describe the patterns of biological signals [5,8] in terms of dynamic approach, such as the Fractal Dimension (FD) analysis. In general, FD can be used to quantify the complexity of an object. In the peculiar case of the CoP trajectory, a change in fractal dimension may indicate a change in control strategies for maintaining quiet stance [5]. Previous studies [5,7] concluded that fractal analysis represents a reliable method to highlight specific characteristics of balance control. Doyle et al. [5] assessed the reliability of traditional and fractal dimension measures of quite stance CoP in young healthy individuals. They demonstrated that although traditional measures are used extensively to assess CoP, their reliability is questionable. On the contrary fractal dimension measures show promise to reliably quantify CoP. Blaszczyk et al. [9] used fractal dimension technique in healthy elderly individuals with their eyes open and closed. Their results evidenced that a change in fractal dimension was representative of a change in stability and balance. Some applications can be found in the literature on gait [10-14] and recently this technique was applied during walking comparing stride-to-stride variability in treadmill walking vs. overground walking [15]. To our knowledge, most of the analyses have been performed only on healthy individuals. Only one study was conducted in Parkinson and ataxia patients [6]. The authors found that the fractal dimension was more sensitive than traditional stabilometric analysis in the evaluation of postural instability. In addition, these studies were generally conducted using fractal dimension approach in one dimension (for postural analysis in the anterior-posterior and medio-lateral direction, separately); no bi-dimensional analyses have been conducted.

According to these studies, the postural pattern can be assessed more quantitatively by computing FD. In pathological conditions, this method has been proven to be useful in evaluating postural instability in Parkinson and ataxia, also adding further parameters to the traditional methods [6]. In addition, FD has been shown to be an excellent measure of quite stance CoP under a number of conditions as compared to the traditional ones [5].

Prader-Willi Syndrome (PWS) is a chromosomal disorder characterised by obesity, muscular hypotonia, ligament laxity and mental retardation. In this condition, movement and postural disorders are common and tend to progressively worsen as the clinical picture advances, severely limiting the patients' quality of life.

Based on the encouraging application of FD approach on healthy subjects and Parkinson patients, our aim was to quantify postural strategy in PWS, not only considering the CoP trajectory analysis in time and frequency domain, but also applying the FD technique. A deeper understanding of the postural abnormalities in PWS may improve the definition of rehabilitation planning and treatment. In the literature, the two studies previously conducted on PWS patients, used time domain parameters only [3,16].

## Methods

### Participants

We enrolled 11 adult patients (5 males, 6 females; age: 34.4 ± 3.7 years) affected by Prader-Willi Syndrome (PWS), who were periodically hospitalised at the San Giuseppe Hospital, Istituto Auxologico Italiano, Piancavallo (VB), Italy.

All patients showed the typical PWS clinical phenotype [17]. Cytogenetic analysis was performed in all participants; 10 had interstitial deletion of the proximal long arm of chromosome 15 (del15q11-q13). Moreover, uniparental maternal disomy for chromosome 15 (UPD15) was found in 1 female. All PWS subjects showed mild mental retardation. In this respect, one of the requirements for participating in the study was a score over the cut-off value of 24 in the Mini Mental State Examination (MMSE) Italian version [18]. Scores over this cut-off are commonly interpreted as absence of widespread acquired cognitive disorders in adult people. All PWS patients were able to understand and complete the test.

Twenty age-matched individuals (10 males, 10 females; age: 31.4 ± 9.6 years) were included as controls (Control Group: CG). Exclusion criteria for the CG included prior history of cardiovascular, neurological or musculoskeletal disorders.

All participants were free from conditions associated with impaired balance, vision loss/alteration, vestibular impairments, neuropathy, as detected by the clinical examination, intracranial hypertension. The study was approved by the Ethics Committees of the Institute. Written informed consent was obtained by the parents or, when applicable, by the patients.

### Experimental set-up

Static posturography was conducted on a non movable force plate (Kistler, CH; acquisition frequency: 500 Hz). The participants were asked to stand for 30 seconds on the force platform, their feet placed with an angle of 30° and their arms at their sides. The individuals are instructed to maintain normal standing balance, undisturbed stance with eyes opened looking at a black target 1.5 m far away (a circle with a diameter of 6 cm) which was positioned vertically to be in the patient's direct line of sight. To avoid any kind of learning or fatigue effect [19] only one trial was acquired.

The test was verbally requested by the same experimenter without providing any modelling or prompting instructions. If the subject was not able to execute the action on verbal request, additional help was given in the following order: (1) verbal prompting: cues and hints; (2) modelling prompt: action first demonstrated by the operator (i.e. "Watch me, look in front of you the black target, please maintain the arm at your side, ...").

### Data analysis

The outputs of the force platform allowed us to compute the CoP time series in the A/P direction (CoPAP) and the M/L direction (CoPML). The first 10 s interval was discarded in order to avoid the transition phase in reaching the postural steady state [2]. The output of the platform was processed to compute quantitative parameters in time and frequency-domain as well as using fractal dimension technique. In particular the following parameters were considered:

### Time-domain parameters

The antero-posterior and medio-lateral coordinates of the CoP trajectory underwent a post-acquisition filtering using a low-pass filter with a cut-off frequency of 10 Hz [20]. As for time-domain analysis, the following parameters were identified and computed:

• RANGE: the range of CoP displacement in the A/P direction (RANGEAP index) and the M/L direction (RANGEML index), expressed in mm;

• Sway Path (SP): the total CoP trajectory length, expressed in mm.

All parameters were normalized to the participant's height (expressed in meters), according to literature [21], in order to avoid the influence of different subject's height on the results and to their foot length (expressed in millimeters); in fact, short feet are one of the typical features of PWS [17].

### Frequency domain parameters

With regard to the frequency analysis of the postural sway, the signals were firstly down-sampled (anti-aliasing filter) at 10 Hz. The analysis was performed using parametric estimators based on autoregressive (AR) modelling of the data [2]. In this study we considered the following frequency-domain parameters:

• the centre frequency of the main spectral peak of the Py spectrum (fy);

• the centre frequency of the main spectral peak of the Px spectrum (fx).

### Fractal Dimension

FD was computed on the image of CoP trajectory using the box-counting method [22]. Briefly, let's superimpose a square grid on the image, being ε the edge size of each square, and let's indicate as N(ε) the number of squares needed to fully cover the image. It can be shown that, in the limit ε → 0 we have

(1) $$\text{N}\left(\varepsilon \right)\sim 1∕\varepsilon^{\text{D}}$$

where D is known a box-counting fractal dimension. The quantity D can be estimated by computing N(ε) for different values of grid size ε. According to relation (1) this yields an array of points in log-log space that can be fitted with a straight line whose negative slope provides an estimate of the FD value. This parameter allows estimating the stabilometry pattern more quantitatively than the traditional methods. A FD in a two-dimensional picture ranges from 0 to 2, with 0 for the point, 1 for the straight line and 2 for the plane. This value is higher when the picture is more complex.

### Statistics

All the previously defined parameters were computed for each participant and then the mean values and standard deviations of all indexes were calculated for each group. PWS and controls' data were compared using Mann-Whitney U tests, in order to detect significant differences. Null hypotheses were rejected when probabilities were below 0.05.

## Results

The clinical characteristics of PWS and CG are reported in Table 1. Age was not significantly different among groups. BMI, weight and height in PWS group were significantly different from CG.

**Table 1:** Clinical characteristics of the study groups (PWS: Prader-Willi Syndrome; CG: Control group)

	*PWS GROUP*	*CG*
Participants (M/F)	11 (5/6)	20 (10/10)

Age (years)	34.4 (3.7)	31.4 (9.6)

Height (cm)	150.6 (6.8)*	173.3 (5.1)

Weight (Kg)	93.9 (18.6)*	62.6 (8.5)

BMI (Kg/m^2^)	41.4 (8.1)*	22.8 (3.2)

Foot length (mm)	207.9 (9.1)*	239.9 (11.4)

Data are expressed as mean (standard deviation). * = p < 0.05, PWS GROUP versus CG.

### Time domain parameters

The analysis of CoP vs. time confirmed previous founding [3] and showed that in PWS participants all the parameters were statistically different from CG, with greater displacements along both the antero-posterior and medio-lateral direction (RANGEAP and RANGE ML parameters). In addition SP parameter was longer if compared to CG (Table 2).

**Table 2:** Postural parameters of the study groups (PWS: Prader-Willi Syndrome; CG: Control group)

	*PWS GROUP*	*CG*
***Time domain***		

RANGEAP (1/m)	0.07 (0.02)*	0.02 (0.01)

RANGEML (1/m)	0.07 (0.03)*	0.03 (0.02)

*TL (mm/m)	3.53 (1.57)*	0.85 (0.99)

***Frequency domain***		

fx (Hz)	0.09 (0.09)	0.16 (0.16)

fy (Hz)	0.14 (0.09)	0.12 (0.15)

***Fractal dimension***		

FD	1.58 (0.08)*	1.12 (0.08)

Data are expressed as mean values (standard deviation). The values of the time domain parameters are normalised for the subject's height and foot length.

### Frequency domain parameters

As for the frequency analysis, our data showed that no significant differences were found between PWS and CG, in both antero-posterior (Px parameter) and medio-lateral (Py parameter) directions, evidencing that PWS and CG use the same frequency in posture maintenance (Table 2).

### Fractal dimension

FD parameter evidenced that PWS were characterised by greater values in comparison with CG. While CG displayed a signal with a fractal dimension close to 1, PWS were characterized by a higher fractal dimension value, indicating a more complex and irregular signal over time (Table 2).

## Discussion

PWS is a complex multisystemic disorder with an incidence of 1/25.000 live births [23] characterized by muscular hypotonia, ligament laxity, hyperphagia, severe obesity, short stature, hypogonadism, mental retardation and dysmorphic features. Both hypotonia and excessive body weight may affect the development of motor and functional skills of PWS individuals which are characterized by postural instability and a cautious abnormal gait [3,24-26]. The characterization of postural capacity appears a key element for depicting the functional profile of the PWS population, which is known to have poor balance and greater risk of fall than healthy individuals. As the literature on this topic is scanty and researches have been conducted only using the traditional methods in time domain, the aim of this study was to analyze the postural control in PWS individuals using not only posture analysis in time domain but also applying the frequency domain analysis and the FD method. With this approach we aimed to investigate whether this new analyses of the dynamics of the CoP movement could add clinically useful information. Traditional measures of CoP in time domain, such as the range of sway and the total trajectory length measured during quite stance, have, in fact, shown poor reliability [5]. Despite universal acknowledgement and a wide use in the clinical practice, they should therefore, to some extent, be cautiously interpreted. FD analysis of CoP has previously shown excellent reliability [5] and, according to this study, can be considered a reliable measure of CoP during quite stance.

Our analysis was conducted first using the traditional method with time (the range of sway in antero-posterior and medio-lateral direction and the total trajectory length) and frequency domain approaches and then integrating them with the FD approach.

As for time domain, our data are in line with a previous study [3,16,27] showing that PWS patients are characterised by higher values of CoP excursion in both antero-posterior and medio-lateral direction with longer CoP trajectory as compared to healthy controls. While the differences in the antero-posterior direction have been attributed to the activation of the ankle plantar flexors/dorsiflexors motor control and to the increased muscular activity with lower motor control present in these patients [28], in the medio-lateral direction the greater CoP displacements are probably related to the loading/unloading mechanism [28] and they involve specific mechanisms operating at the hip level rather than the ankle muscle control [29].

Frequency analysis showed that PWS patients displayed the same frequency of controls, even if the range of motion is higher in all directions. Since frequency parameters are related to the velocity at which the CoP moves, these results could underline that the changes in time domain did truly reflect the impairment in postural control, rather than a different strategy adopted by PWS. This kind of analysis adds information to the traditional parameters, analyzing the rate at which the CoP direction changes, reflecting the action-reaction times between external perturbations and compensatory movements in order to reestablish balance. Time domain parameters are in fact, according to some researchers [20,30,31], not sufficient for the detection of early changes in standing balance. The stabilogram, in fact, has dynamic characteristics and posture must be considered as dynamic stability of a continuously moving body, also characterized by chaotic fluctuation of CoP trajectories. These elements are not detected by time domain analysis of CoP; on the contrary, frequency-domain characteristics and dynamical system theory seem to be more appropriate for characterizing posture and can more likely allow for the detection of early changes in the system function [32]. The commonly used method to describe posture in the frequency domain is the non-parametric method, which utilizes the Fast Fourier Transform. When dealing with pseudo-stochastic signals, the use of parametric power spectrum estimators, such as those based on AutoRegressive models of the data which we used in our analysis, may have some advantages, especially when short data segments are available and few harmonic components have to be retrieved from a wide-band noise [33].

As for the FD approach, our results showed that PWS were characterized by higher values of FD parameter when compared to CG. These values are indicative of the complexity of the stabilometric pattern in PWS individuals in postural maintenance. The non-linear approach takes in account the dynamic of the signal. The higher FD values in PWS may also be interpreted as an inability of those patients to synergically modulate the three systems (i.e., visual, vestibular and somatosensory) involved in maintaining posture. Our body is continuously exposed to external perturbations, which we try to counteract by integrating the real-time inputs and the prediction system based on previous inputs: the information given by the non-linear approach can well describe this mechanism. Our data reflect the difficulties encountered by PWS in adapting to this process. Recently, Cimolin et al. [27] demonstrated in fact that PWS patients are characterised by unchanged postural stability when eyes are open and closed, showing that balance in PWS is not influenced by visual input. They assumed that proprioception is prevalent over visual input in the development and setting of postural control system in PWS. Such anomalous modulations of the systems involved in balance maintenance are confirmed by the result obtained using FD approach.

This study showed that the FD adds information to the traditional time and frequency domain analysis of the CoP in individuals with PWS, providing a more informative description of their posture.

The main limit of this study is the small number of enrolled participants which results in limited strength of the clinical and statistical findings. In addition, as overweight is a distinctive feature in PWS, the analysis should have been more rigorously compared with obese instead of normal-weight individuals. In this way, in fact, we were not able to isolate the effects of obesity and those directly connected to the genetic disorder in terms of postural instability. However, our investigation represents a preliminary attempt to integrate traditional posturographic methods with the FD analysis of CoP during quiet stance in a pathological condition characterized by reduced balance capacity. Further studies should be conducted to confirm these data considering larger groups of patients with other balance disorders.

## Conclusion

In this study we investigated whether the FD approach would add relevant information to the traditional analysis of the CoP trajectory (time analysis) using static posturography in individuals with PWS.

Our data demonstrated that the analysis in the frequency domain did not seem to explain the postural deficit in PWS, as their values are close to controls. Conversely, the FD method appears to provide a more informative description of it and to complement the time domain analysis.

## Competing interests

The authors declare that they have no competing interests.

All authors attest and affirm that the material within has not been and will not be submitted for publication elsewhere

## Authors' contributions

All authors read and approved the final manuscript.

VC made substantial contributions to data interpretation and was involved in drafting the manuscript

MG made contribution to conception, design and interpretation of data, revising the manuscript critically and gave the final approval of the manuscript

CR made contribution to data analysis and interpretation

GG made contribution to interpretation of data, revising the manuscript critically

LV made substantial contributions to data acquisition, elaboration and interpretation

LM made contribution to data interpretation, revising the manuscript critically

PC made contribution to conception, design and interpretation of data, revising the manuscript critically and gave the final approval of the manuscript
